# A Life Cycle for Modeling Biology at Different Scales

**DOI:** 10.3389/fpls.2021.710590

**Published:** 2021-09-03

**Authors:** Kate Harline, Jesús Martínez-Gómez, Chelsea D. Specht, Adrienne H. K. Roeder

**Affiliations:** ^1^Section of Plant Biology, School of Integrative Plant Science, Cornell University, Ithaca, NY, United States; ^2^Weill Institute for Cell and Molecular Biology, Cornell University, Ithaca, NY, United States; ^3^L.H. Bailey Hortorium, Cornell University, Ithaca, NY, United States

**Keywords:** evo-devo, biomechanics, morphodynamics, developmental modeling, plant morphogenesis

## Abstract

Modeling has become a popular tool for inquiry and discovery across biological disciplines. Models allow biologists to probe complex questions and to guide experimentation. Modeling literacy among biologists, however, has not always kept pace with the rise in popularity of these techniques and the relevant advances in modeling theory. The result is a lack of understanding that inhibits communication and ultimately, progress in data gathering and analysis. In an effort to help bridge this gap, we present a blueprint that will empower biologists to interrogate and apply models in their field. We demonstrate the applicability of this blueprint in two case studies from distinct subdisciplines of biology; developmental-biomechanics and evolutionary biology. The models used in these fields vary from summarizing dynamical mechanisms to making statistical inferences, demonstrating the breadth of the utility of models to explore biological phenomena.

## Introduction

Biological systems represent nested hierarchies of complex patterns and processes. Models have arisen as a tool to reveal salient aspects of these systems (May, 2004). Models help expose the function of complex systems: For example, by representing processes on the sub-phenotype scale, models can reveal emergent properties of a system such as tissue patterning that occurs despite local cellular variability (Hong et al., 2016). Modeling can also reveal the source of counterintuitive effects; for example, a gene promoting expression of its own negative regulator to obtain stable expression in a feedback loop (Alon, 2007; Ding et al., 2020). Complementarily, models can use statistical inference to discover patterns in large datasets like inferring evolutionary phylogenies or gene regulatory networks (Beaulieu and Donoghue, 2013).

Modeling and computational literacy have become ever more pressing skills as high-throughput methods of data acquisition become more accessible. With modern datasets getting ever larger, biologists have turned to advanced statistical methods and modeling approaches to process and interpret these large datasets.

Modeling biological systems requires extensive background in mathematics, statistics, programming, and biology. As most researchers fall somewhere within the wide spectrum of skill levels in each of these fields, it can be difficult for a single individual to efficiently and correctly obtain the biological information about a question, formalize the question into rigorous mathematics, and apply appropriate statistics. Interdisciplinary collaborations with mathematicians and statisticians are an invaluable solution to this lack of knowledge. However, careful communication must be maintained between collaborators to make sure the original biological question does not become lost in the details (Bak-Maier and Inger, 2007; Palmer, 2018; Kluger and Bartzke, 2020). Biologists are often competent users of specific programs that model processes of interest with a general understanding of how these models relate to others, yet knowledge gaps remain that can lead to the misinterpretation of the model or a lack of knowledge of field standards for applying certain formalisms. It can be argued that a general level of understanding is sufficient. However, as the use of models to describe biological mechanisms becomes more pervasive, all biologists must move to improve their competencies to critically analyze models and ensure that the assumptions they, a collaborator, or other researcher are applying are sound (Makin and Orban de Xivry, 2019). The time necessary to properly implement a model and interpret its results relative to the biological question can be daunting. Nevertheless, it is necessary to prevent improper use of statistical methods and modeling approaches, which can diminish their utility or worse lead to misinformed conclusions.

Motivated by this need, we take a bird’s-eye view and present a blueprint for the life cycle of a biological model. While previous reviews have focused on specific mathematical models (Bartocci and Lió, 2016), the overall goals of biological modeling (Sharpe, 2017) or specific biological processes to model (Bucksch et al., 2017), we instead highlight the logic behind biological abstraction and how a model and its development can be used to refine hypotheses. While non-exhaustive, our framework focuses on how to formalize biological hypotheses into mathematical language, and how to design data driven experiments to test these hypotheses. By putting forward these basic tenets of modeling, we hope this manuscript serves as a guide for researchers to apply models to their own investigations and to critically assess the validity of models in the literature.

We use the two case studies that follow to demonstrate the utility of this blueprint. The first case study details a model that attempts to predict the change of a system over time from a set of initial conditions, while the second case study is concerned with statistical inference of present states due to past events. In order to establish the broad applicability of this blueprint, we source these examples from two distinct fields of biology; developmental biomechanics and evolutionary biology. These fields are historically distinct and concerned with different levels of biological organization and different temporal scales: the development of individual organs vs. the macroevolution of species. Despite the distinct research programs and models applied, they both can be examined under our unifying blueprint. By demonstrating how the principles of our life cycle can be adapted and reinterpreted at two distinct spatial and temporal scales, we hope readers can infer how to approach biological modeling problems at many scales.

## The Modeling Life Cycle

**Figure 1:**
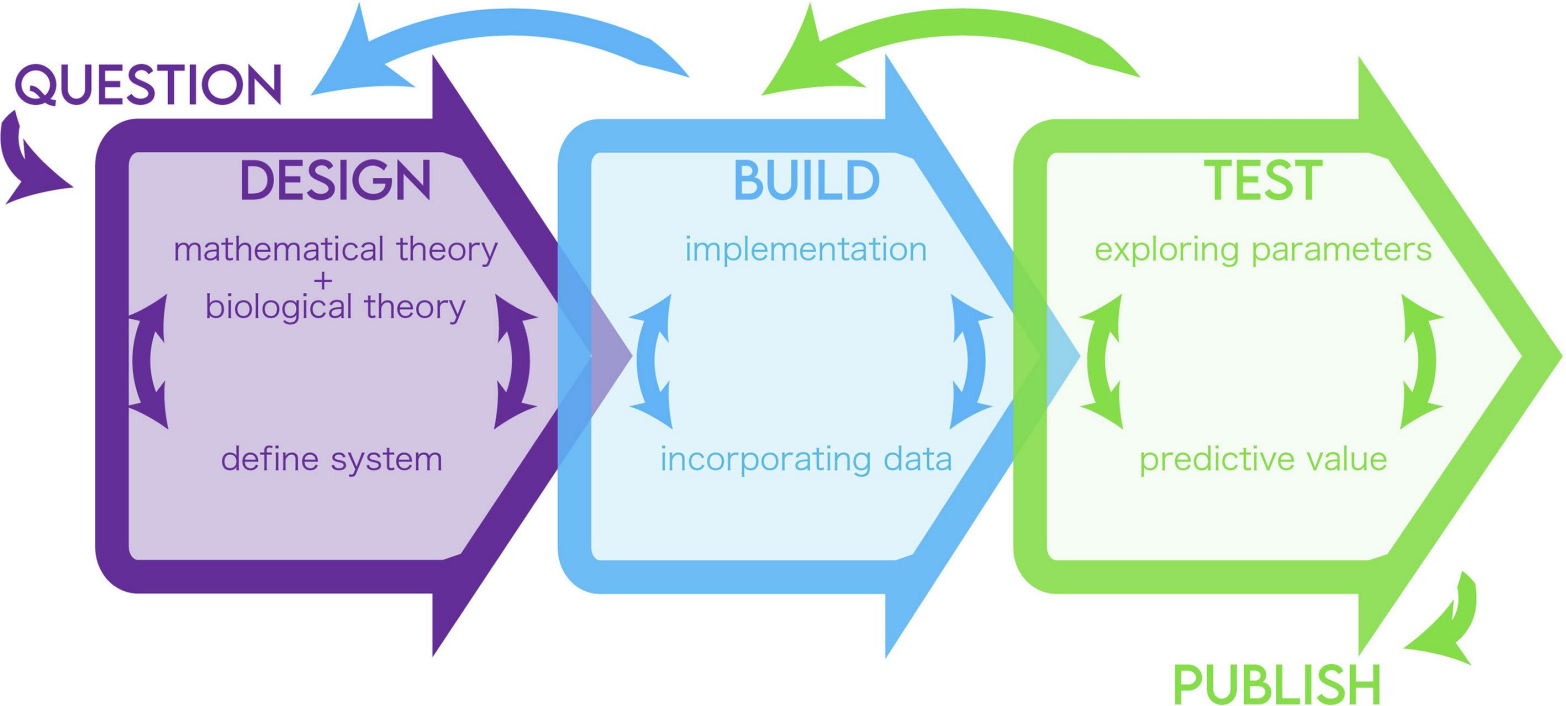
Summary of the modeling life cycle.

Here, we highlight a general blueprint for modeling biological systems (Figure 1). We draw the basic tenets of “design, build, and test” from the field of engineering and elaborate on how they can be broken down into steps of a modeling pipeline. We define design as the definition of the biological problem and its translation into formulas, build as the selection of methods to solve the formulas, and test as the analysis of the solution and results in relation to the modeler’s goals. These stages are fluid and likely interchangeable, but we chose this framework to suggest breakpoints in the process where concordance between the question, the model, and the modeler’s goals can be evaluated. We aim to illuminate key areas to troubleshoot in a developing model where there is a disconnect between the information used and the question being asked. The cycle may also help validate and explain key, unintuitive insights of the model. In the same way that in a lab, the experimental system must be matched to the question and the question refined to fit within the confines of experimental resources, so too must the theory and implementation of a model be refined. We hope that tracing a model along this framework will offer the researcher, reviewer, or reader a flexible and iterative guide to assess models as well as to build their own.

### Pose Biological Question

Not all biological questions require modeling approaches. Often much can be figured out through descriptive “models.” For example, classic “models” of plant development like the ABC model of floral morphogenesis and the CLAVATA-WUSCHEL feedback did not require computational investigation to uncover the fundamental mechanisms governing these phenomena (Coen and Meyerowitz, 1991; Somssich et al., 2016). However, our understanding of even these systems has benefitted from computational models. Formal gene regulatory models allowed researchers to investigate the molecular dynamics underlying the descriptive models (Espinosa-Soto et al., 2004; Jönsson et al., 2005; Alvarez-Buylla et al., 2008; Gordon et al., 2009; Chickarmane et al., 2012; Zhou et al., 2018; Refahi et al., 2021). A biologist must critically assess whether new insights will be gained from modeling. Determining whether or not a model will contribute new insights relevant to a particular hypothesis or set of hypotheses depends on what data are available, and what remains unknown or unsolvable. Models are well-suited for questions where connections between well-studied inputs and outputs, key regulatory hubs in complex systems, or patterns in data are not clear from empirical observation alone. Models also may assist in choosing between hypotheses by rapidly testing what combination of relationships or system characteristics (parameters and topologies) do or do not approximate empirical results. Models are also helpful if they can offer quantitative predictions (Ellner and Guckenheimer, 2011; Transtrum and Qiu, 2016). The modeler must decide which type of question they would like to investigate, and if they have (or can obtain) enough data to fit and test the model (see Incorporating Data).

## Design

The goal of model design is to explicitly define the biological hypothesis and formalize it into mathematical equations.

### Biological Theory

With a biological question in mind, the modeler must enumerate the known biological inputs, outcomes, and relative spatial and temporal scale at which these relationships are predicted to occur. It can be helpful to summarize these details into a diagram indicating the hypothesized interactions between inputs and outcomes. This information will help determine the definition of states and the equations linking the states together. The modeler should decide which temporal scale(s), spatial scales, biological relationships, and characteristics they want the model to represent and what evidence they have for identifying these factors. This will be used in analyzing the fit of the model (see Test: Predictive Value) and choosing which simplifications and assumptions can be made (see Build: Implementation). For dynamic models, modelers should have an idea of the spatial resolution of the interactions they want to study. This will help the modeler represent their system geometrically (see Build: Implementation).

### Mathematical Theory

The biological processes must be converted into a system of equations that can account for the changes exhibited by the biological system. Choosing the form of the equations will impose assumptions on the result that should not be forgotten when interpreting the solution (see Case Studies for examples of valid and overstretching conclusions). For a classically trained plant biologist, this is a great opportunity to explore establishing an interdisciplinary (Palmer, 2018) collaboration or further mathematical training (Bak-Maier and Inger, 2007; Kluger and Bartzke, 2020).

### Define System

Working through biological and mathematical theory should reveal what data can be collected from the biological system to inform the model, what mechanisms and relationships are known to exist, which will be inferred, and what outcome predictions will be tested. This may offer the first opportunity for refining what question the researcher is most interested in and suggest simplifications for other aspects of the system. Classically trained plant biologists may struggle to refine a model because they are used to looking at problems holistically. Whereas physicists and mathematicians are more comfortable knowingly excluding certain aspects of a system in order to study behavior under a particular set of circumstances. This latter approach is not only necessary to enable mathematical implementation, but it also helps to quickly identify areas where a model and its assumptions will no longer apply – and thus where more research, data, and hypotheses are needed. A hybrid approach of different models with different spatial resolutions can provide a helpful link between these two inclinations. Before proceeding to implement the model, the mathematical formulas should be explicitly stated and the extent to which they, and their corresponding parameters, adhere to or abstract from biology should be understood. The system definition locks in the modeling goals, the researcher will be able to achieve. The relationship between the biological and mathematical theory determines the balance of phenomenology and quantitative prediction. This step may become iterative as the modeler balances the complexity of the biological inputs included in the system with the level of mathematical theory, the researcher is comfortable employing.

## Build

The second stage is solving the defined equations, often by implementing the equations into code.

### Implementation

Often to address the specificities of a biological question, it is infeasible to perform calculations by hand. Therefore, most contemporary models require a computer implementation. Computers provide the processing power to handle equation-dense systems and to numerically solve equations that do not allow for a closed form solution (see Box 1: Analytical vs. numerical solutions). Many decisions and tradeoffs must be made at this stage that require knowledge of computer language formalisms, which libraries already exist in a given language and how they are implemented. These implementation details can greatly influence what conclusions and interpretations of the model are valid (Kursawe et al., 2017).

Box 1 **Technical considerations**

Below, we provide a brief summary of some of the technical considerations that are involved in the implementation of a model. Further detail is beyond the scope of this review as it concerns a deeper dive into the fields of mathematics and computer science.

*1) Analytical Vs. Numerical Solutions*

Analytical solutions are possible when the equations can be rearranged to solve for a variable of interest. This is possible if the functions defining a system are only dependent on one variable (position, time, etc.). Or if certain parameters would be orders of magnitude less than others, their influence can be ignored as negligible and the equations simplified to the aforementioned form (Alon, 2007; Ellner and Guckenheimer, 2011). When analytical solving is not possible, a numerical method is employed. For example, in calculus, this is the estimation of an integral through summing the area of small rectangles under a curve. The numerical approximation chosen determines a certain level of over- or under-estimation error that will be accepted in the solution (Hairer et al., 1993). For models with multiple continuous variables, a discretization method must be selected to obtain a numerical solution. Discretization provides rules for how the continuous aspects of a model will be broken into smaller problems for solving. For example, to discretize time, the time step and equations to be solved at each time step must be defined. To discretize space, a geometry (line, plane, surface, etc.) must be chosen and broken up into distinct parts (line segments, triangles, etc.). Choosing the numerical and discretization methods can significantly impact the results of modeling so the researcher should be able to justify their decisions by extensive literature search or support from work with experienced collaborators. Some researchers may want to begin by stating their problem in terms of discrete mathematics in order to avoid the need to discretize later. This may make the implementation more straightforward as the discrete equations can directly translate into code. In either case, the researchers still should analyze the solution in areas of parameter space deemed biologically relevant to ensure the implemented solution upholds biologically prescribed behavior. These exercises are often referred to as “sanity checks.” Sanity checks can include ensuring solutions converge where expected, analyzing regions where parameters are asymptotic (get very big or very small), and checking assumptions using analytical simplifications to solve the equations under conditions that can be measured empirically. When analyzing discrete-time models, it is important to use time scales much larger than the time step used in implementation.

*2) Implementation Strategy*

In the simplest case, where a researcher would like to apply a previous model to a new system, they may be able to utilize code or a graphical user interface (GUI) as-is, with minimal additional programming or a few parameter adjustments. However, as the technical details of a model diverge more from existing implementations, the researcher must become better versed in the programming languages and libraries available to answer their questions and which underlying assumptions are hard-coded or parameter-adjustable. The former might entail using the classic spring model to represent a cell wall, but only changing the spring constant to match a measurement of cell wall resistance to growth (elasticity, or stiffness) to obtain a simple force relationship calculation. Whereas, the latter, more intensive implementation might arise from a desire to model the forces experienced by each cell in a tissue. This could be accomplished by specifying behavior (e.g., a field of biomechanical relationships) for a computational structure (e.g., a mesh; see Case Study I).

*3) Computational Complexity*

Another consideration in implementation is that the computer solves the problem in such a way that the number and types of simulations that are required will not be so computationally expensive as to become time or cost prohibitive (e.g., determining the number of iterations and when results are logged; Arora and Barak, 2009). This can be influenced by the type and structure of data, the researcher supplies to the pipeline (e.g., processing of strings vs. lists requires different amounts of compute-time based on the way they are stored and accessed in memory; Cormen, 2009).

### Incorporating Data

A defining part of building a model is the integration of real data. Data may be used to inform how model parameters are set and thus limit the range of outcomes. Here, it is important that modelers and scientists are on the same page about what has physically been measured and how directly that relates to parameters in the model. Parameter values can be fixed by summarizing collected data using statistics (e.g., average, median etc.), or, probability distributions for collected data can be sampled during simulations. Measurements may also be combined and incorporated into a model as single parameters. Oftentimes ratios that combine measurements turn out to be key parameters in determining a process (Munoz and Ortega, 2019). For a few examples of experimental strategies to collect data relevant to model parameters, see Table 1. If a large enough dataset can be collected, it can be helpful to separate the data into testing and training sets. The training set will be used to fit model parameters, while the testing set is set aside to ensure that the trained model is predictive (see Test: Predictive Value). Often models can contain many more parameters than is practical or feasible to measure. At this point, the researchers must decide what simplifications or assumptions they will make about parameter values to solve the model.

**Table 1:** Examples of biological questions to model and typical approaches for mathematic representation and data collection. Other probabilistic approaches can be added to account for randomness.

Type of question	Mathematical approach	Data collection
Regulatory networks, population dynamics	Kinetics, logic circuits, and differential equations (Alon, 2007; Ellner and Guckenheimer, 2011)	Field measurements, photobleaching and recovery, pulse-chase experiments, and FRET (Meyvis et al., 1999; Bunt and Wouters, 2004; Ellner and Guckenheimer, 2011; Simon and Kornitzer, 2014)
Morphogenesis	Mechanics, physics, and differential equations (Niklas, 1992; Boudaoud, 2010; Howard et al., 2011; Shapiro et al., 2012)	Atomic force microscopy, live imaging, osmotic treatments, and other turgor measurements (Milani et al., 2013; Beauzamy et al., 2014; Weber et al., 2015; Kierzkowski et al., 2019)
Phylogenetic reconstruction, network inference, Motif identification	Markov chains, statistical hypothesis testing, tree manipulations, and graph theory (Jukes and Cantor, 1969; Felsenstein, 1985; Friedman, 2004; O’Meara, 2012)	Character matrix scoring, fossil traits and associated dates, and DNA alignments (Löytynoja and Goldman, 2005; Iles et al., 2015)
Pattern formation	Reaction diffusion equations, feedbacks, and bistability (Murray, 1982; Howard et al., 2011)	Photobleaching and recovery, pulse-chase experiments, and imaging (Meyvis et al., 1999; Bunt and Wouters, 2004; Simon and Kornitzer, 2014; Sapala et al., 2018; Ding et al., 2020)

## Test

Once a model has been implemented, the model must be tested for functionality and utility.

### Exploring Parameters

Certain parameters inevitably cannot be measured because it would be technically challenging or time intensive to do so (Huang et al., 2018; Munoz and Ortega, 2019). Based on the measurements that can be made, these parameters must be explored and set within a range that creates biologically relevant model outputs. When no measurement has been taken or the researcher wants to widely vary the value of a parameter, the researcher may simply choose a set of numbers for a given parameter (i.e., designating a parameter as being composed of only integers or all Real numbers). The field of parameter estimation provides rigorous methods for testing parameter sets to fit a model to data (see Box 2: Parameter Estimation).

Once a model has been produced with “reasonable” parameters, the relative influence of each parameter on a model’s output can be tested to reveal key steps in a process. The parameter space should be explored by modifying parameters one at a time and in different sets across many orders of magnitude. Parameter space exploration will point out which parameters must remain relatively stable, and which can be allowed to vary and still produce similar simulations (Murray, 1982; Huang et al., 2018; Ren and Murray, 2018). Parameters that can be varied over many orders of magnitude without significantly changing the model output may represent sources of robustness in a system. Robust components allow organisms to develop reliably in changing environments because the outcome of a process can remain stable even given variable inputs (Nijhout, 2002; Abley et al., 2016).

Parameter sensitivity analysis is another parameter exploration strategy that asks how much a model output depends on a given parameter. This can reveal which motifs, rates, or transitions are key elements underlying a particular pattern or process and therefore cannot change without impacting the system. Parameters with high sensitivity may represent good targets for manipulation when the goal is to change a phenotype (Hamby, 1994; Cho et al., 2003) or evolutionary outcome (Wheeler, 1995).

For machine learning models, data visualization strategies have been developed to probe the aspects of input data that are captured by different model features (Samek et al., 2017).

Box 2 **Interpretation standards**

Choosing a final model to summarize or predict a process is a balance of accuracy and simplicity. Models can be tested for their utility and accuracy using statistical methods, summarized below.

*1) Parameter Estimation*

The goal of parameter estimation is to obtain values for each parameter in a model by fitting experimentally derived inputs and outcomes. For example, calibrating branch lengths in a phylogeny to the fossil record will limit the possible rate of speciation by bounding (constraining) time (Steel et al., 1996). In another case, setting a parameter to the measured degradation rate of a protein or signaling molecule could set a realistic range for its rate of diffusion from a modeled source (Levine et al., 2007; Howard et al., 2011).

Parameter estimation is achieved by minimizing a cost function that calculates the difference between the model prediction and the measured outcome, given the parameter values used. Parameter values are changed until a reasonably small value is obtained for the cost function (Ellner and Guckenheimer, 2011; Myers, 2018). The likelihood is a common cost function. The likelihood of a model calculates the probability that the data collected could have been produced from the parameters, given the model. Recent work has shown that often many parameter values are “sloppy.” That is, they can be allowed to vary over large ranges without having a significant impact on the output of the model. Meanwhile, a few “stiff” parameters have much larger impacts. By analyzing the relative “sloppiness” of parameter sets, it may also become apparent that it is not individual parameters, but specific composites (ratios etc.) of parameters that create “stiff” regions in the model (Brown et al., 2004). The SloppyCell framework provides methods to analyze the “sloppiness” of parameter sets in dynamic models (Gutenkunst et al., 2007; Daniels et al., 2008). A new model refinement method has been proposed to use the discernment of these “stiff” parameter spaces to remove or merge these “sloppy” parameters (Transtrum and Qiu, 2016).

*2) Model Selection*

Comparison between similar models can help select the best model based on the modelers’ goals. The likelihood cost function can be used to compare models. Likelihoods can be compared by likelihood ratio tests. Programs can be told to examine different models until a minimum improvement in likelihood is achieved.

Models may also be selected based on their simplicity, i.e., the minimum number of parameters that can provide valid and insightful predictions. The field of information theory offers a few solutions for selecting models by penalizing the number of parameters they contain. A common strategy is computing the Akaike Information Criterion (AIC; Johnson and Omland, 2004; Arnold, 2010; Neath and Cavanaugh, 2012).

Where quantitative prediction is the goal, “usefulness” is best quantified using a testing set of data. The test set is a subset of the data set aside before fitting the model. It provides a complete set of inputs and outcomes and therefore can statistically quantify the validity of the prediction made. A testing set will help determine if overfitting has occurred. Overfitting is when the model has been fit to noise in the training data set and cannot make inferences if provided new input data (Hawkins, 2004; Srivastava et al., 2014; Makin and Orban de Xivry, 2019).

### Predictive Value

Assessing the predictive value of a model requires that the researcher revisit their biological question and modeling goals. These goals will determine how the accuracy of model outputs over the interpretability of individual model states or parameters is weighed. If the modeler is trying to make quantitative predictions, they will need to undergo more rigorous statistical analysis of the model outputs compared to measurements. However, the underlying components of the model may be allowed to stray more from biological principles. A “black box” model, where the internal configuration of the model is determined more by statistical fit than biological principles, may be acceptable if it can achieve quantitative prediction goals, however, it is less valuable for describing phenomenological underpinnings. If the modeler is more interested in understanding conceptually how a biological system works the qualitative reproduction of states may be more important, while the quantitative outputs of the model are less important (Ellner and Guckenheimer, 2011; Transtrum and Qiu, 2016). In this case, simulations can be designed to visually present model states and outcomes for qualitative comparison to the data. The internal structure of the model will also be more important to the researcher in this case. It will be more important that functions contain plausible biological relationships. With either goal in mind, most modelers seek the simplest representation of their system – with as few parameters as possible (see Box 2: Model selection).

## Case Studies

We offer two case studies below to guide the reader through the life cycle of a model. Each case study will provide concrete examples of the basic principles outlined in the life cycle, taking the reader directly from principles to practice. Most of what we describe can be found in the Materials and Methods or Supplementary Information provided with the cited texts. However, in certain cases, we made assumptions based on field standards or conferral with the authors. While our life cycle aims to encompass the entire development of a model, from first principles to final simulation, often a single publication (including ours selected) does not explicitly cover each of these stages. Therefore, in the analysis of the case studies, we elaborate on how the theoretical context and prior advancements enabled the advancement of a new model, beyond what is explicitly covered within the paper.

The first case study focuses on the interplay between genetic and biomechanical factors in floral organ development (Hong et al., 2016). The second case study focuses on the study of fruit evolution using models to make inferences regarding timing and order of evolutionary events (Beaulieu and Donoghue, 2013).

## Developmental Biomechanics

Development is the study of the progressive deployment of genetic programs to create organisms. To move beyond description and test the dynamical connections between genetic players and their biomechanical outcomes, mathematical models must be employed. The following example comes from the field of computational morphodynamics. Computational morphodynamics investigates the relationship between genetic components and the biomechanical properties of cells (Chickarmane et al., 2010; Roeder et al., 2011). A few recent examples include investigating developmental identity domains, organ polarity, and the mechanics of leaf and floral organ morphogenesis (Hamant et al., 2008; Kuchen et al., 2012; Abley et al., 2013; Skopelitis et al., 2017, 2018; Fox et al., 2018; Kierzkowski et al., 2019). In the paper, detailed in this case study, the authors used this integrative strategy to explore how stable organ size and shape arise in nature.

Organisms have a surprising ability to establish body plans with organs of a standard size, despite the noisy environments in which they develop. As noted in the paper, intuition would predict that organs of reproducible size and shape would result from equivalent growth rates and directions amongst their cells (Hong et al., 2016). However, imaging living organs over many days revealed notable variability among the rates and directions of cellular growth (Tauriello et al., 2015). The authors turned to modeling to reveal the unintuitive leap necessary to understand how an organ could develop at a standard size despite underlying stochasticity in growth amongst its cells.

### Pose Biological Question

How does stable organ size emerge? How is spatially and temporally variable growth at the cellular level coordinated toward this goal? Testing the biomechanical properties of tissues requires methods that lead to tissue deformation and destruction, but measuring development requires that the tissues of interest remain unmanipulated and undamaged during organ growth. Thus, modeling was also necessary to test the link between biomechanic inputs and the outcome of growth that the authors predicted from their experiments.

## Design

### Biological Theory

**Figure 2:**
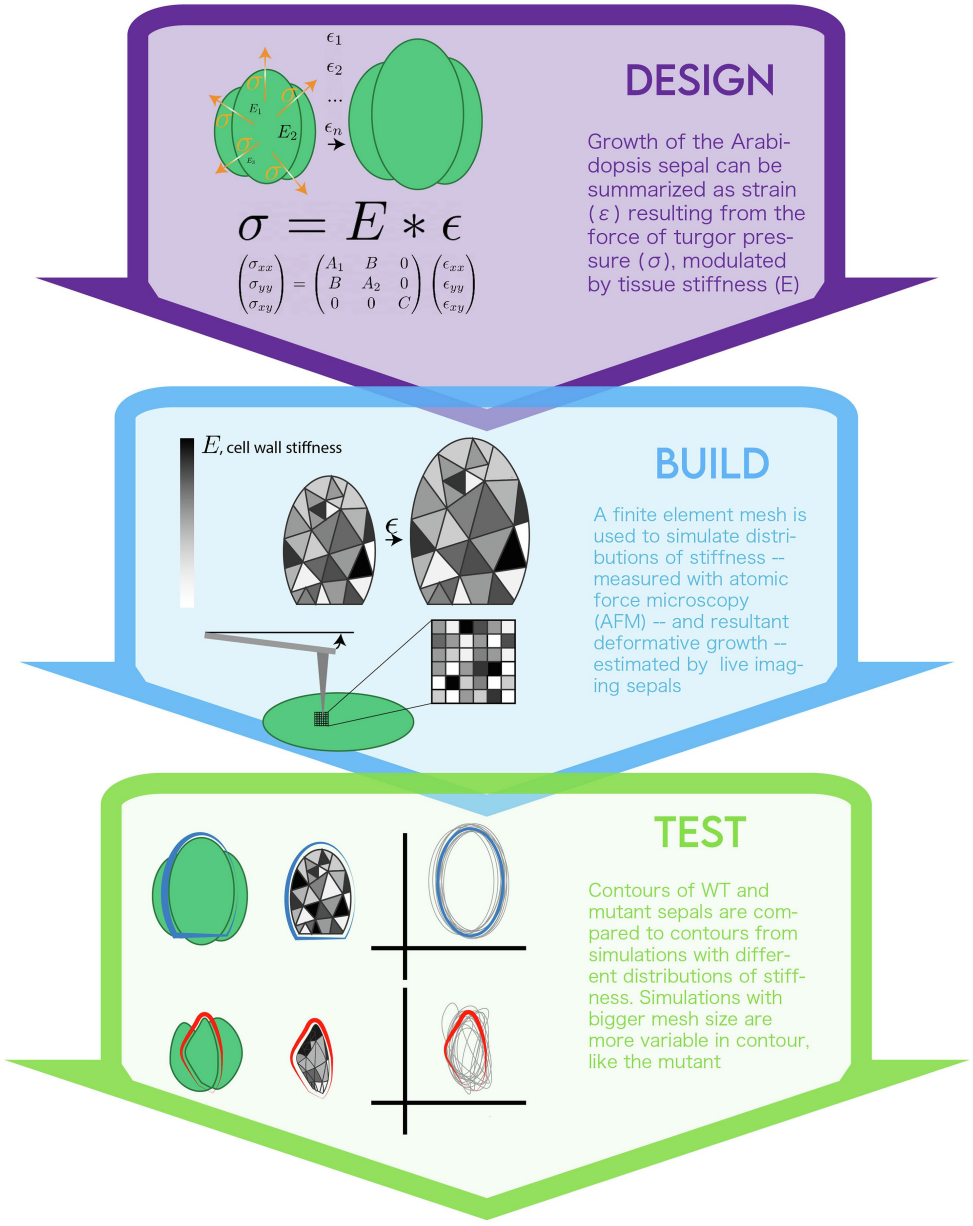
The modeling life cycle used to implement a biomechanical model of sepal growth.

The authors chose to investigate the development of sepals in the *Arabidopsis* flower to discover mechanisms of organ size and shape regulation. A mutant screen for sepals with unusually high levels of size and shape variability identified the *vos1* (*variable organ size and shape*) mutant. A standard *Arabidopsis* flower initiates four small bulges along the perimeter of the bud to begin making sepals. These bulges then grow to enclose the bud, each obtaining an approximately elliptical shape. Unlike in wildtype flowers, in *vos1* mutants, each of the primordial bulges grows to form sepals with different disjointed shapes, and the internal floral organs become exposed as the sepals fail to enclose the flower bud. The authors noticed a difference in the “correlation length” between *vos1* vs. WT sepals whereby cell wall stiffness was less variable across space in *vos1* mutants than in WT flowers (Figure 2). This prompted the hypothesis that the different stiffness distribution between WT vs. *vos1* mutants was impacting growth (due to different yielding of cell wall material to turgor pressure) and thus causing the variable organ development phenotypes. Since the cell wall properties can be variable within an organ in both space and time, the authors aimed to develop a model that could represent growth and stiffness in independent subregions within the sepal.

### Mathematical Theory

Because this problem involved complex spatial and temporal information, the authors turned to a system of dynamic differential equations to simulate their system. These equations are based on the classic relationship expressed in Hooke’s Law. Hooke’s Law (*F* = kx) was originally used to define the displacement (x) of a spring by application of a given force (F). It can be adapted to any system in which there is a linear relationship between a force applied and the deformation of a material. If the problem is defined as discrete in time, Lockhart’s equations,Eϵ=σ, for the force of turgor pressure against plant cell walls applies (Lockhart, 1965). In this relationship, sigma is the turgor pressure (i.e., stress), E is the resistance to permanent deformation (i.e., stiffness) and epsilon is growth in the unit of time defined by the discretization (i.e., rate of strain). For more on applying mechanics to plant morphogenesis (see Boudaoud, 2010).

### Define System

The authors modified an existing model of yeast growth to account for the starting and final geometry of a sepal (Bonazzi et al., 2014). The sepal primordium was defined as a semi-circle. The system of Lockhart equations was formulated in two dimensions so that the planar (*x*,*y*) deformation of the initial semi-circle could be tracked.

A few assumptions were made in the implementation of this model. Most notably, it was assumed that turgor pressure is homogenous in plant tissues and adhered to previous measurements (Beauzamy et al., 2014). It was also assumed the indentation achieved with atomic force microscopy (AFM) was indicative of cell wall stiffness. AFM measures the amount of force required to displace a cantilever a certain distance (Kirby, 2011). Recent work has demonstrated the importance of AFM indentation depth in measuring local vs. global material properties of a tissue (Long et al., 2020). Further, it was assumed that two dimensions are sufficient to summarize the growth of a sepal. In other words, the influence of cell layers and differences between epidermal and internal tissues is negligible. Recent work has shown the importance of moving toward modeling inner cell layers to account for organogenesis (Zhao et al., 2020) By using a simpler representation of the organ, the authors were able to model organogenesis on a larger time scale. Many biomechanical models do not attempt to summarize this biological time. They typically feature more of the biological reality (and therefore more computational complexity) by providing rules for cell division and by including more genetic regulatory inputs (Kuchen et al., 2012; Fox et al., 2018; Kierzkowski et al., 2019). Yet, this level of information limits the amount of time that can be represented before computational resources become limiting. While there is a tradeoff in the level of detail, the model represents along this larger time scale, this model has the benefit of being able to probe development continuously from primordia to final organ, rather than merely representing a few developmental stages as in others.

## Build

### Implementation

Differential equations in multiple dimensions (*x*-*y* space and time) cannot be solved analytically in general. The finite element method was chosen to discretize the problem and yield a numerical solution (see Box 1: Analytical vs. Numerical Solutions). *Via* this method, the initial sepal shape was subdivided into a mesh of interconnected triangles, known as “elements.” Six “elements” were approximated as representing one cell. It was decided that this scale would allow the level of spatial variability within and between cells to be captured, without making the simulations too computationally expensive. The modelers varied growth rates over space and time by assigning each element a stiffness, E, at each step. The size of the mesh, i.e., the number of elements, was varied between simulations to mimic differences in the “length” (level) of correlation between stiffness of local regions. The mesh size therefore was used to test the spatial variability. With stiffness and turgor pressure set, Hooke’s law was solved to determine how the mesh representing the organ would deform at each step of the simulations. The model then “grew” the mesh shape to this new solution, and stiffness values for each element were recalculated based on this deformation. The program ran until a user-ascribed size was reached or other execution conditions were met.

Code was written in C++ to interface with the FreeFem++ finite element solving language and allow the user to control the number of iterations and output files produced (Hecht, 2012). Output files of stiffness distributions, tissue-wide growth rates, and other model parameters were processed using Python scripts.

### Incorporating Data

By imaging the same plant organ (the *Arabidopsis* sepal) every day, every 12 h, the authors could measure growth at the cellular level (represented by epsilon, strain in the equations; de Reuille et al., 2015). These measurements were taken over the course of early to late development, so that the model’s deformation could be fit to this progression of states. Sepal geometry changes were factored into the model by taking many measurements of the contours of sepals and measuring the points on the perimeter of the simulation meshes. The variability of simulation contours was then fit to the variability of sepal contours. AFM and osmotic treatments were used to measure the stiffness of the cell walls (E in the Lockhart equation; Milani et al., 2013). Turgor pressure was assumed to be uniform across the organ and its value based on previous work (Beauzamy et al., 2014). Given these biological measurements, the other unitless weights in the expanded planar form of Lockhart’s equation were tuned so the final mesh contour would achieve approximately the same ellipsoidal shape of a WT sepal. The variability of the final shapes from simulation and experiment were analyzed by applying Fourier methods. Fourier methods are a common mathematical method used to solve equations, and in this case represent the shapes of the contours and thus allow for their comparison. Scripts similar to those used for the model outputs were developed to process biological sepal shape contour data so that the impact of variable stiffness on growth in simulations and experiments could be compared rigorously.

## Test

### Parameter Exploration

WT plants seem to have quite a bit of spatial and temporal variability in cell wall properties and growth, which allows them to reproducibly yield stable organ size and shape. A parameter sensitivity analysis was conducted to ask: how does stiffness varying in space and time yield stable organ shapes? Exploring the parameter space by varying the mesh size showed at what point the relative local variability was large enough to simulate sepals with *vos1* phenotypes. WT observations were more consistent with simulations that included smaller mesh sizes.

### Predictive Value

The differences between WT and mutant populations obtained by experiment and model simulation were compared using permutation tests. Permutation tests indicate whether the statistics of populations are the same, agnostic of the underlying distribution of the datasets. The model was deemed accurate for its ability to recapitulate the statistical level of variability between the shape of WT and *vos1* contours. The authors mostly aimed to have a qualitative representation of the underlying biology and were able to tie mechanisms implemented in the model to empirical measurements, so they did not rely on a quantitative output of the model to determine its predictive value.

## Summary

The simulations showed that given spatial mechanical variability, temporal mechanical variability allows the production of standard organ shapes. When the stiffness could not vary enough in space, the sepal grew into odd and unnatural shapes, like *vos1* mutants that do not perform the sepal function of protecting the bud. Therefore, the authors concluded, the key determinant of a sepal obtaining a final standard size was the maintenance of stiffness variability in space. This model was useful because it introduced new questions to the field: over what time spans is variability important? What signals help enforce the variability and act on timing? The flexibility of the basic model allowed the authors to continue adjusting biomechanical inputs and simulate new mechanisms of growth regulation. This model shed light on the flexibility of development to handle stochasticity in environmental conditions (Debat and David, 2001; Lempe et al., 2013). The aspects of growth that were not probed in this model leave room for the expansion of these ideas to account for cellular divisions and multiple cell layers.

## Evolutionary Biology

An integral part of evolution is understanding the order of trait evolution (e.g., ancestral vs. derived). Assessing this is a challenge as the ancestral conditions that gave rise to the diversity of present day species and their traits occurred millions of years ago, leaving us with only traces of past processes. Thus, models of evolution must use evolutionary theory to incorporate data from modern species, fossil data if available, and phylogeny, to infer diversification events in the past. The interpretation of these models forms the basis of comparative developmental biology as they allow us to infer homology (Wake et al., 2011; Church and Extavour, 2020). In the following example, we discuss the logic and assumptions of an evolutionary model of fruit evolution (Beaulieu and Donoghue, 2013). The methodology employed by the authors is commonly termed ancestral state reconstruction (O’Meara, 2012). The authors were interested in investigating the evolution of fruit-type as this morphological trait can determine the dispersal ability of the species of interest. Dispersal distance (dispersibility) is positively-correlated with diversification potential since this distance determines the probability of establishing new populations distant from the parent, increasing opportunities for establishment, and filling new niches while reducing competition from the parental plant.

### Pose Biological Question

Many different questions regarding the evolution of fruit can be answered from such an approach. One question the authors ask is: what was the ancestral fruit state of the Campanulids (the clade including sunflowers, carrots, honeysuckles, and relatives)?

## Design

### Biological Theory

The group of study, the campanulids, is a diverse group of plants with variation in fruit-type. Historically, the fruits of campanulid species have been separated into four botanical types – drupe, berry, capsule, and achene. However, the authors reclassified the fruit type based on three binary (coded 0 or 1) traits: dehiscent or indehiscent, single- or multi-seeded, and dry or fleshy. The use of binary traits helped clarify the typological classifications that have the tendency to cluster phenotypes based on single traits (for example; you can have a dry or a fleshy berry and these might have different developmental origins). Additionally, they enabled the possibility of exploring two fruit types that are not readily classified into canonical fruit types: indehiscent, dry, and multi-seeded (IDM) and dehiscent, dry, and single-seeded (DDS).

This model is based on the assumption that the exhibited traits are heritable and their modifications are passed between generations, eventually spawning distinct species with distinct fruit types (Darwin, 1876).

### Mathematical Theory

The authors chose to use a continuous-time Markov process to account for the evolutionary transitions between the combinations of fruit-type binary pairs; an approach commonly used to infer ancestral condition of discrete traits (Pagel, 1994). A markov chain represents evolutionary changes in fruit-type as a directed graph that summarizes the transitions (i.e, graph edges) between discrete states (i.e., graph nodes) with certain probabilities (i.e., weights on graph edges). Probabilities of transition (aka transition rates) represent the expected amount of changes between two states for a given phylogeny. As data for extant organisms are located at the tips, transition rates are dependent upon relative ages of divergence represented in the phylogeny. The Markov chain is summarized using a “Q matrix,” in which the rows and columns reflect the different states and matrix values represent the transition probabilities between the respective row-and-column states. In order to fit the model, a maximum likelihood framework is used to estimate the parameter values (e.g., transition probabilities and ancestral states) that best explain the data. To do this, a modification of the Felsenstein pruning algorithm is used to calculate the likelihood of the data (see section “Incorporating data” below) given a set of parameter values (Felsenstein, 1973; Pupko et al., 2000). The algorithm uses fruit type data for species at the tips, the phylogeny tree structure and a given set of transition probabilities to guide the stepwise calculation of each ancestral state at the speciation events in the phylogeny (i.e., nodes in the phylogeny). A heuristic search algorithm is used to find the set parameters values that maximize the likelihood for the entire tree. While this study used a maximum likelihood framework as a goodness-of-fit criterion to fit parameter values, other frameworks can be used such as parsimony and bayesian (Swofford and Maddison, 1987; Huelsenbeck and Bollback, 2001). Continuous time markov model can be used to model the evolution of any discrete traits, including for nucleotide substitution used for phylogenetic inference (O’Meara, 2012).

### Define System

**Figure 3:**
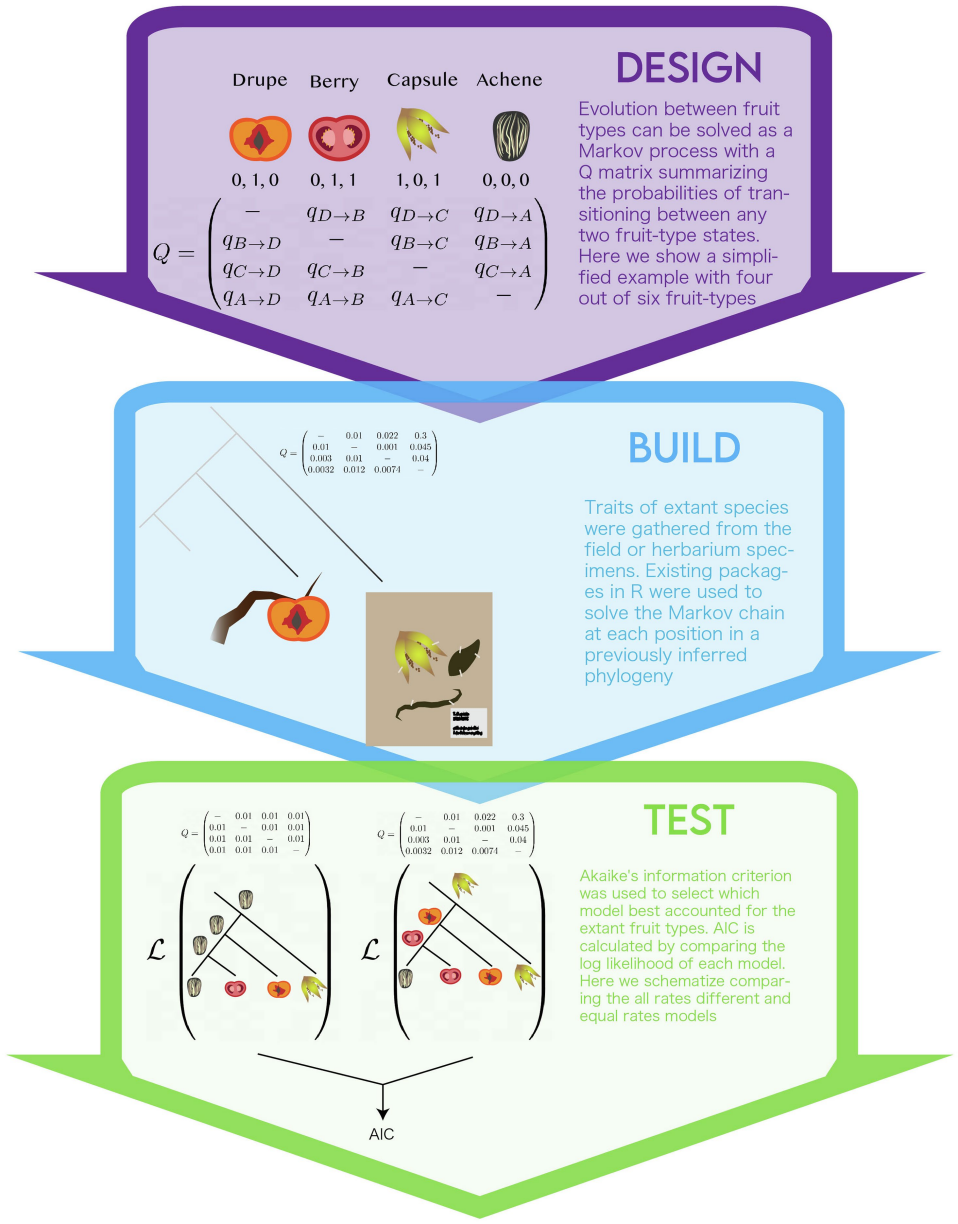
The modeling life cycle used to implement the multistate model of the evolution of fruit-type.

The authors model the shift of character states at the scale of morphological fruit-types (Figure 3). Some genetic information is retained as the phylogenetic tree is inferred from genetic sequence. The authors defined two different Q matrices to summarize transitions between fruity types. The first defined transitions between all six combinations of their binary traits observed in extant species, termed the “multi-state” model (e.g., drupe, berry, capsule, achene, IDM, and DDS). This model required multiple changes between fruit types, so the authors set up another Q-matrix, termed the “correlated paths” model. In this model, only one state out of the three binary characters is allowed to change for a given transition, therefore, all transitions represent one evolutionary change. The authors were concerned about the artifacts that might arise in calculating transition probabilities for states that are not measured in extant species. Based on genetic evidence they accepted the assumption that these states were not possible and omitted them from the “correlated paths” model. The model also assumes that diversification rates did not co-vary with fruit-type. In other words, if a particular fruit type influences speciation and/or extinction rates, as predicted by evolutionary theory, this influence would not be modeled (Maddison, 2006). Other assumptions stem from the phylogeny. The authors used a fully bifurcating phylogeny, this disregards evolutionary processes, such as incomplete-lineage sorting and hybridization, non-bifurcating speciation events, and extinction that may have shaped the evolution of fruit type. Inference of these factors in phylogenies remains its own challenge. Development of coherent models of trait evolution for non-bifurcating phylogenies remains an active area of research (Bastide et al., 2018).

## Build

### Implementation

Ancestral state inference by continuous time markov models has been implemented in a number of software packages (Pagel et al., 2004; Paradis et al., 2004; Revell, 2012; Maddison and Maddison, 2019). The authors used a software package they developed for the statistical language R, called corHMM (Beaulieu et al., 2013). This package provides users with the ability to specify a number of models, the method used to infer the root (Yang, 2006; FitzJohn et al., 2009) and whether the rest of the tree is solved *via* joint, marginal, or scaled approaches. Interested readers are encouraged to refer to the materials and methods of the paper or R documentation for details.

### Incorporating Data

The two types of data the authors used are the fruit type characters for all species studied and molecular data for all species in the phylogeny. The authors used a previously published phylogeny inferred by aligning 12,094 nucleotide sites from 8,911 extant species. The sites consisted of sequences from chloroplast genes and nuclear ribosomal Internal Transcribed Spacer regions (Beaulieu and Donoghue, 2013). This set of genes allowed for the largest species sampling available at the time. The authors felt confident in this phylogeny’s representation of the evolution of campanulids because it was consistent with relationships recovered from many other phylogenies derived from traditional character-scoring methodologies and including varied taxonomic sampling of the campanulid lineage. Fruit-type data for each of the extant species were scored for the three binary traits either from samples collected in the field, herbarium specimens, or published accounts of the species (i.e., species descriptions).

## Test

### Exploring Parameters

Unlike models of extant plant morphology (see Case study I) in which the model parameters can be directly compared to a real plant organ, the evolutionary history of transitions in fruit morphology across species is not observable. As such, model selection, a method to compare the parameters of competing models, was used. The authors used the AIC as a measure of model fit (Akaike, 1998). AIC weighs the log likelihoods of a model by the number of parameters, this way it accounts for overparameterization, and models of different parameters can be directly compared. The authors calculate AIC for two variations of the “multi-state” and “correlated paths” models, each under two assumptions: that either all the transition rates were the same – “equal rates model”– or with all transition rates different – “all rates free.” In this way, they compare four different models of trait evolution.

### Predictive Value

Again, as inferences of the model cannot be directly observable, it is a challenge to determine whether these results predict natural phenomena. One possible source of validation is if a fossil is discovered that confirms the results of these models: however, associating a fossil with a particular extant clade has its own challenges (Steel et al., 1996). A slight variation is to simulate multiple datasets under the best fit model and test if some salient aspect of observed data is reflected in the distribution of the simulated datasets. This is termed model adequacy (Pennell et al., 2015), here, fruit types are simulated using the Q-matrix of the best fit model and a t-test is used to assess if the observed data (i.e., extant species fruit) reflects simulated data. For example, we can measure the frequency of fruit type for extant taxa (e.g., 10% capsule), simulate data, and check if the simulated dataset has a similar distribution. The study at hand did not do this but the authors have in subsequent publications (Beaulieu et al., 2013).

## Summary

Using this approach, the authors find that the “correlated paths” model with all rates free for transitions had the best fit (i.e., lowest AIC). Under this model, the ancestral condition of the campanulids is a capsule. Interestingly, this model predicts zero transitions from single to multi-seededness fate and from any other state to capsule. These zero transition rates offer fascinating hypotheses about the directionality of evolution, indicating that while all transitions are theoretically possible, they are not observed in this group. Further, some of the highest transition probabilities were associated with the evolution of the achene fruit-type, which matches well with the overall abundance of this fruit-type represented in the clade. Their model assumes that the evolution of this trait is only governed by a transition probability but note their conclusions may be confounded by the fact that a large group with the achene fruit-type exhibited much higher rates of diversification than clades bearing other fruit types. A class of models, the species-dependent speciation extinction (SSE) models jointly account for both transition rates and diversification rates associated with a character state (Maddison, 2006). The authors did fit a SSE model, the Binary SSE (BiSSE) model, which they used to show that species with achenes have higher rates of diversification compared to species with other fruit types (Maddison et al., 2007) BiSSE can only model a character with two character states (i.e., achene vs. non-achene). More complex SSE models that allow for greater than two character states (e.g., MuSSE) were in their infancy at the time (FitzJohn, 2012). Explicit definition of the model assumptions will allow these data to be explicitly integrated with new data and analyzed with advancing methods in future studies.

## Conclusion

In this review, we provide a framework to guide the development and interrogation of biological models. We acknowledge that the process of modeling often will not neatly fit into the compartments we have provided. However, we hope that the compartments that we provide help modeling paper readers, reviewers, and writers better question their understanding of a given model and interrogate its value in answering a given biological question.

Determining the value of a model is a difficult and at times contentious exercise (Prusinkiewicz and Coen, 2007). The outcome depends on the modelers’ goals (which may differ greatly from the expectations of a reader or reviewer). Typically, the goals of modeling are either to (a) use abstraction and refinement to get to the simplest explanation of a process and often at the expense of strict adherence of parameters to biological analogs or (b) incorporate all known biological inputs and attempt to recapitulate a general behavior or system property, at the expense of a comparable numerical output.

Authors should strive to be explicit in the publication what the goals of the model were to temper the interpretation of quantitative vs. qualitative results for their audience. Authors could explain how it is sufficient to publish a “black box” model by proving it to be highly predictive of an important input-output relationship. Similarly, authors can justify the inability of a model to necessarily match measurements if the model can qualitatively recapitulate observable biological behaviors with model components that have biological analogies (see Box 3: What to Publish). Properly contextualized models will guide research forward by illuminating “control knobs,” for a process, general design principles of a system and/or by providing quantitative predictions.

Box 3 **What to publish**

When a model is ready for publishing, it is critical to detail experimental materials and methods such that readers can reproduce the presented results. Reviewers and readers should have a clear explanation of the theoretical equations and the implementation of their numerical approximations. Modelers should strive to adhere to high standards of code readability and availability. Just as raw data should be made available, code should be either provided in the supplemental information or hosted in a virtual repository and the link provided with the text (free versions exist for GitHub, Bitbucket). For models of biochemical processes, the Systems Biology Markup Language (SBML) offers notable portability of models between analysis software (Hucka et al., 2004). Modelers can specify their models in this format or use SBML tools to read/write their models in/out of various network analysis tools. This provides the opportunity for easier sharing of model specification while allowing end users to choose in which programming environments they want to explore the model (Hucka et al., 2004). For model implementations, where SMBL is not applicable, modelers might want to implement a command-line interface or GUI. This would allow researchers without familiarity with a given language to input their data and parameters through passed arguments or computer dialog screens without requiring the underlying code to be edited. When models are easier to use and share, science progresses faster because hypotheses can be retested in different biological systems.

## Author Contributions

KH and JM-G proposed and developed the ideas underlying the manuscript and contributed equally. All authors contributed to the writing of the manuscript.

## Conflict of Interest

The authors declare that the research was conducted in the absence of any commercial or financial relationships that could be construed as a potential conflict of interest.

## Publisher’s Note

All claims expressed in this article are solely those of the authors and do not necessarily represent those of their affiliated organizations, or those of the publisher, the editors and the reviewers. Any product that may be evaluated in this article, or claim that may be made by its manufacturer, is not guaranteed or endorsed by the publisher.
